# Supplementary material for: MetaRibo-Seq measures translation in microbiomes
Source: Nat Commun. 2020 Jun 29;11:3268. doi: 10.1038/s41467-020-17081-z (PMC7324362; doi:10.1038/s41467-020-17081-z)
Supplement: Supplementary file 10 — Supplementary Data 7 [file 41467_2020_17081_MOESM10_ESM.zip › File2/Confidence_High_Taxonomy/312090_out.krona.html]

Javascript must be enabled to view this page.

members
magnitude
magnitudeUnassigned
count
unassigned
taxon
rank

312090\_out

34

2
34
superkingdom

34
1239
phylum

186801
34
class

34
186802
order

species

SRS042966\_contig\_number\_17938SRS043768\_contig\_number\_contig-100\_9988.9989SRS045004\_contig\_number\_32622SRS045645\_contig\_number\_10359SRS045713\_contig\_number\_21481SRS050422\_contig\_number\_21185SRS050752\_contig\_number\_24884SRS050925\_contig\_number\_36853SRS054059\_contig\_number\_4389SRS054905\_contig\_number\_6437SRS058723\_contig\_number\_4047SRS063190\_contig\_number\_contig-100\_3450.76050SRS063489\_contig\_number\_7878SRS076756\_contig\_number\_21856SRS077335\_contig\_number\_7759SRS077552\_contig\_number\_contig-100\_3737.137366SRS078242\_contig\_number\_31264SRS078419\_contig\_number\_contig-100\_425.197298SRS098881\_contig\_number\_19616SRS101376\_contig\_number\_28372SRS1041031\_contig\_number\_30152SRS1041140\_contig\_number\_10713SRS104400\_contig\_number\_50615SRS104693\_contig\_number\_8587SRS1055069\_contig\_number\_10649SRS142890\_contig\_number\_contig-100\_24718.127019SRS143417\_contig\_number\_25275SRS144135\_contig\_number\_36474SRS146764\_contig\_number\_22465SRS146812\_contig\_number\_58989SRS147346\_contig\_number\_56271SRS147614\_contig\_number\_6109SRS148424\_contig\_number\_34623SRS893383\_contig\_number\_31262
1897047
34
